# The Impact of Psychological Health on Patient Recovery After Arthroplasty

**DOI:** 10.3389/fpsyt.2022.817716

**Published:** 2022-06-30

**Authors:** Zhen Zhang, Qiqi Xing, Da Zhong, Yixiao Pan, Tailai He, Yihe Hu, Long Wang

**Affiliations:** ^1^Department of Orthopedics, Xiangya Hospital, Central South University, Changsha, China; ^2^Hunan Engineering Research Center of Biomedical Metal and Ceramic Implants, Xiangya Hospital, Central South University, Changsha, China; ^3^Department of Orthopedics, First Afliated Hospital, School of Medicine, Zhejiang University, Hangzhou, China; ^4^Hunan Key Laboratory of Aging Biology, Xiangya Hospital, Central South University, Changsha, China; ^5^National Clinical Research Center for Geriatric Disorders, Xiangya Hospital, Central South University, Changsha, China

**Keywords:** hospital anxiety and depression scale, psychological health, total joint arthroplasty, postoperative recovery, postoperative satisfaction

## Abstract

**Purpose:**

The purpose of this study was to determine the relationship between psychological health and postoperative recovery and satisfaction in patients undergoing total joint arthroplasty (TJA).

**Methods:**

We prospectively enrolled patients undergoing TJA from July 2019 to December 2020. A psychological evaluation was conducted according to the Hospital Anxiety and Depression Scale (HADS). Based on the preoperative HADS scores, we grouped the patients into two groups: the symptomatic group and the asymptomatic group. Data on the Harris Hip Score (HHS), Knee Society Knee Scoring System (KSS), Forgotten Joint Score-12 (FJS-12), Short Form-12 (SF-12), and Numeric Rating Scale (NRS) for pain in these two groups were collected preoperatively and postoperatively. Then, these data were analyzed by Statistical Package for Social Sciences (SPSS) version 19.

**Results:**

The final cohort consisted of 80 patients. Patients undergoing TJA had significantly decreased HADS and NRS scores and improved HHS, KSS, SF-12, and FJS-12 scores (all *p* < 0.001). Compared with the symptomatic group, the asymptomatic group showed better postoperative recovery (*p <* 0.05), especially after total knee arthroplasty (TKA) (*p <* 0.05). Good postoperative recovery positively impacted the patients’ postoperative psychological state.

**Conclusion:**

Finally, the psychological state can affect recovery after TJA, and successful TJA can help improve patients’ psychological states, especially after TKA.

## Introduction

Total joint arthroplasty (TJA) is the most effective treatment for end-stage osteoarthritis, rheumatoid arthritis, and other joint diseases; it has been proven to reduce patients’ pain and improve patients’ joint function and quality of life (1, 2). Although TJA has achieved great success, not every patient is satisfied with the postoperative results (3–6). There is a known association between prolonged severe pain and an increased risk of psychological distress (7). Patients who need to undergo TJA may suffer considerable pain in the long term, which increases their potential risk of psychological distress. Psychological disorders can negatively impact the way that a person feels, acts, and thinks; therefore, psychological disorders may negatively influence patient satisfaction and functional outcomes related to treatment.

Some studies (8–12) have demonstrated that psychological state influences TJA outcomes, whereas other studies (13–16) have found no significant correlation between preoperative mental health status and postoperative physical function. Therefore, whether a psychological state can affect postoperative recovery is controversial.

This article focuses on people of Asian descent who are undergoing TJA, and the purposes of this study were to explore the dynamic correlation between psychological state and postoperative recovery: (1) to investigate the influence of the preoperative psychological state on early-term postoperative recovery and satisfaction in patients and (2) to discuss the impact of postoperative recovery on the postoperative psychological state of patients.

## Materials and Methods

### Study Design and Data Sources

This was a single-center prospective cohort study involving patients undergoing TJA from July 2019 to December 2020 at Central South University Xiangya Hospital. No patients had been treated and all patients were classified into two groups by the Hospital Anxiety and Depression Scale (HADS) score. We compared the differences between these two groups by observing and analyzing postoperative recovery indicators. Ethical approval was granted by the Ethics Committee of Central South University Xiangya Hospital, and the trial was registered at ClinicalTrials.gov (ChiCTR1900023061).

### Inclusion and Exclusion Criteria

The inclusion criteria were as follows: (1) patients with knee or hip diseases, including osteoarthritis, femoral head necrosis, rheumatoid arthritis, and traumatic arthritis; (2) patients who had exhausted conservative treatment; (3) patients aged between 18 and 75 years; (4) patients with no previous knee or hip surgery; (5) patients who had not been diagnosed with any type of psychological illness prior to inclusion in the study; and (6) patients who provided written informed consent that they were willing to complete all six-month assessments after receiving both an oral and a written explanation of the study protocol. The exclusion criteria included the following: (1) patients who had a cognitive or comprehensive disorder during the study; (2) patients who had any type of psychological disorder and required psychological therapies during the study; and (3) patients who had unsuccessful TJA with surgical complications (clinicians evaluated the success of TJA through unified objective indicators such as postoperative X-ray, blood indicators, and clinical examinations of joint function).

### Outcome Measures

The evaluation of patient satisfaction completely followed the subjective feelings of the patients. Six months after surgery, patients were asked how they felt about the procedure: whether they were satisfied or dissatisfied.

The HADS was used to measure anxiety (HADS-A) and depression (HADS-D) symptoms in psychiatric and medical patients, including patients with the following: chronic pain, spinal cord injury, and rheumatological diseases (17). The HADS is not a diagnostic tool and is a poor predictor for making a specific diagnosis. The score ranges from 0 to 21 (17, 18). The HADS score has been proven by multiple studies (19–23) to be proportional to a patient’s physical and mental state, and the HADS score, as a continuous variable, is positively correlated with the Zung Self-Rating Anxiety/Depression Scale (SAS/SDS) (24). Therefore, this paper used the HADS score as a continuous variable to evaluate the psychological state of patients.

The Reliability and Validity of Sense of Coherence -13 (SOC-13) scale (25) contain three dimensions: meaningfulness, comprehensibility, and manageability (26). The overall scale yields a total score ranging from 13 to 91. Higher scores indicate a stronger sense of coherence. A sense of coherence arises from the salutogenic model of health and reflects a person’s confidence and ability to have a meaningful and manageable understanding of their life (27).

The Harris Hip Score (HHS) was used to assess the clinical health status relevant to total hip arthroplasty (THA) outcomes (28–31). This score includes a rating scale of 100 points regarding pain, function, activity, deformity, and motion. The Knee Society Knee Scoring System (KSS) serves as a scoring system that characterizes the expectations, satisfaction, and physical activities of various patients undergoing total knee arthroplasty (TKA) (32–34), including the KSS-Functional (KSS-F) and KSS-Clinical (KSS-C). In the above two scales, higher scores indicate better status.

The Numeric Rating Scale (NRS) for pain is the favored method of quantifying pain for both clinical and investigational purposes. The NRS score ranges from 0 to 10 points, with higher scores indicating worse pain; patients with higher scores were selected to focus on pain improvement after arthroplasty (35).

The Short Form-12 (SF-12) was selected to evaluate global changes in health-related quality of life. This score ranges from 0 to 100 points, with lower scores indicating worse physical and mental health.

The Forgotten Joint Score-12 (FJS-12) is a valid patient-reported outcome measure used to assess prosthesis awareness during daily activities after arthroplasty. This questionnaire was developed considering that joint awareness is a highly discriminative outcome parameter, especially in patients with good-to-excellent joint function (36). The raw score is normalized to range from 0 to 100 points, and higher scores indicate greater patient satisfaction and better outcomes (37).

### Schedule

**FIGURE 1:**
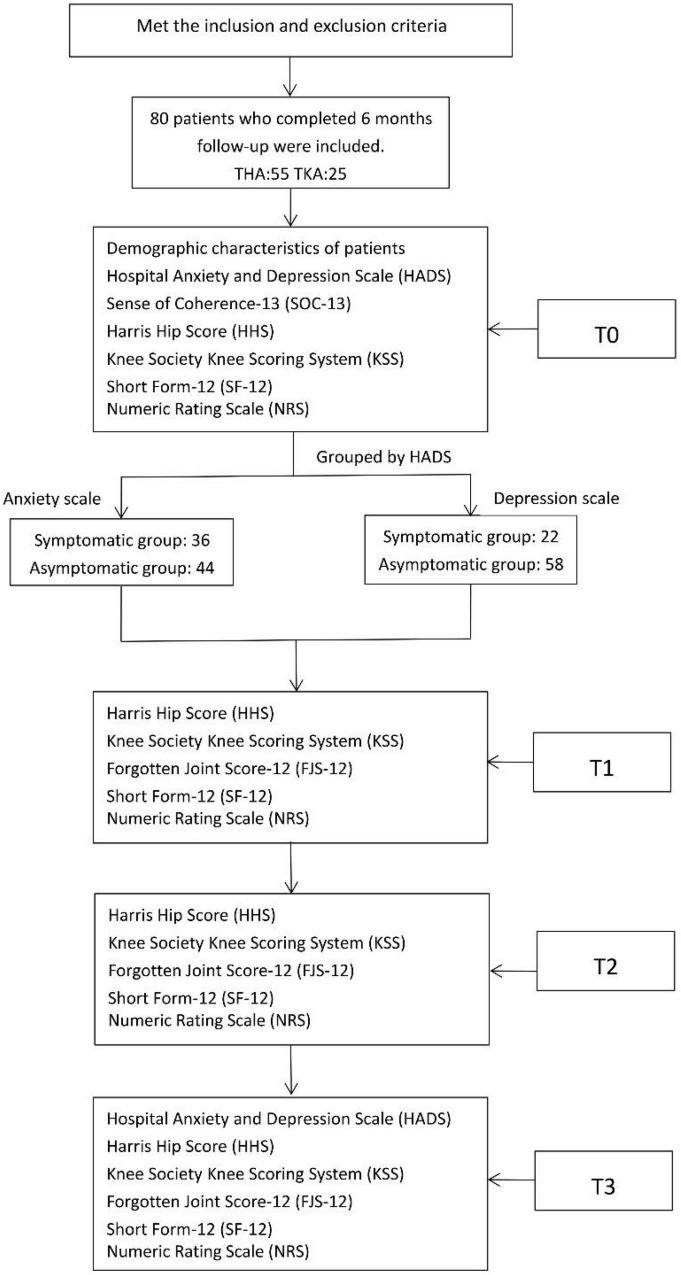
Flowchart for the study design.

Within a week before surgery (T0), the following patient demographic data were collected: age, sex, course of the disease, and body mass index (BMI). Prosthesis types were recorded, and the HADS, SOC-13, HHS, KSS, SF-12, FIS-12, and NRS scores of the patients were evaluated. The HHS, KSS, SF-12, FJS-12, and NRS scores were also evaluated one month (T1), three months (T2), and six months (T3) after surgery. We collected the HADS data and self-administered satisfaction scores (dissatisfied, satisfied) of the patients at T3 (Figure 1).

### Patient Grouping

For descriptive purposes, the patients were divided into groups based on their preoperative HADS scores. The score ranges from 0 to 21, and relevant studies have shown that when the critical score of the HADS is set at 8 points, the HADS has the highest efficiency in identifying patients with anxiety and depression (38, 39). Therefore, we stratified patients into two groups: the symptomatic group (score: 8–21) and the asymptomatic group (score: 0–7) (17, 18).

### Statistical Analysis

Data were collected in excel and analyzed using Statistical Package for Social Sciences (SPSS) version 19 and Prism GraphPad.

Demographic data and other baseline features are described using the mean and the standard deviation for continuous variables; frequencies and proportions are used for categorical variables. Repeated measures multivariate analysis of variance was used to compare the differences between the symptomatic group and the asymptomatic group at different time points. Fisher’s exact test was used to assess whether there was a difference in the disease composition ratio between the symptomatic group and the asymptomatic group. Multivariate linear regression (stepwise method) was used to evaluate the influencing factors of psychological status before surgery and six months after surgery. Independent variables that had no significant effect (*p* > 0.05) on the dependent variable were excluded. *R*^2^ ranges from 0 to 1 and represents the proportion of the change explained by the regression equation in the total change in the dependent variable. The closer *R*^2^ is to 1, the stronger the relationship between the independent variables and the dependent variable. A *p*-value of < 0.05 was considered statistically significant in all analyses.

According to previous studies (5, 6, 40, 41), the satisfaction rates of the psychological disorder group and control group were 60% and 90%, respectively, and a clinically significant effect of 25% or more would be of interest. According to the methods (42) of calculating sample size, we set the beta at a level of 0.20 and alpha at a level of 0.05, and a total of 60–80 patients should be included. The calculation formula is as follows:


$$n=\frac{\left[{\left(a+b\right)}^{2}\left(p_{1}q_{1}+p_{2}q_{2}\right)\right]}{x^{2}}$$

*n* = the sample size in each group.

*p*_1_ = proportion of satisfied subjects in the psychological disorder group.

*q*_1_ = proportion of unsatisfied subjects in the psychological disorder group.

*p*_2_ = proportion of satisfied subjects in the control group.

*q*_2_ = proportion of unsatisfied subjects in the control group.

*x* = the difference the investigator wishes to detect.

## Results

From July 2019 to December 2020, 90 patients were enrolled. Among the 90 patients, 80 (88.89%) were included. Eight patients were excluded because of missed follow-up visits, and two were excluded due to severe postoperative complications. Complete data from 80 patients were collected for statistical analysis; the analyzed cohort included 41 women and 39 men. Twenty-two patients underwent surgery on the right hip, 33 patients on the left hip, 17 patients on the right knee, and eight patients on the left knee.

When the 80 patients were categorized by HADS-A scores, 36 patients were included in the symptomatic group, and 44 patients were included in the asymptomatic group; in addition, they were grouped by HADS-D scores, with 22 patients included in the symptomatic group and 58 patients included in the asymptomatic group. There were no significant differences in age, sex, course of the disease, BMI, prosthesis types, hospitalization time, intraoperative bleeding, length of surgery, or the composition ratio of diseases between the symptomatic and asymptomatic groups (whether grouped by HADS-A or HADS-D scores). The demographic characteristics of the two groups were well matched at baseline (Table 1).

**TABLE 1:** Demographic characteristics.

Characteristic	Anxious		*P*-value	Depressed		*P*-value
	N	Y		N	Y	
Sex (male/female)	24/20	15/21	0.252	29/29	10/12	0.669
BMI	24.27 ± 3.18	23.01 ± 3.01	0.076	23.51 ± 3.27	24.18 ± 2.84	0.403
Age	52.89 ± 12.25	47.50 ± 13.18	0.062	49.50 ± 13.47	53.00 ± 11.06	0.281
Course of Disease (year)	5.55 ± 7.88	5.75 ± 7.58	0.612	6.20 ± 7.85	4.18 ± 7.24	0.236
Hospitalization time (day)	9.23	8.92	0.636	9.31	8.50	0.267
Intraoperative Bleeding (ml)	230.45	219.44	0.783	228.28	218.18	0.821
Length of surgery (min)	101.59	98.94	0.689	100.86	99.18	0.820
Hip/Knee	27/17	28/8	0.115	39/19	16/6	0.636
CR/PS (Knee)	7/10	5/3	0.32	9/10	3/3	0.91
**Hip diseases**						
Osteoarthritis	5	3	0.381	6	2	0.694
Developmental dysplasia	3	6		8	1	
Femur head necrosis	18	16		22	12	
Rheumatoid arthritis	1	0		1	0	
Traumatic arthritis	0	1		1	0	
Hemophilia arthritis	0	2		1	1	
**Knee diseases**						
Osteoarthritis	14	8	1.000	16	6	1.000
Rheumatoid arthritis	1	0		0	1	
Traumatic arthritis	1	0		1	0	
Hemophilia arthritis	1	0		1	0	

*BMI: Body Mass Index; CR: Posterior Cruciate Ligament Retaining Prosthesis; PS: Posterior Stabilized Prosthesis; N: asymptomatic patients; Y: symptomatic patients.*

### Surgical Effect

#### Hospital Anxiety and Depression Scale Scores

**FIGURE 2:**
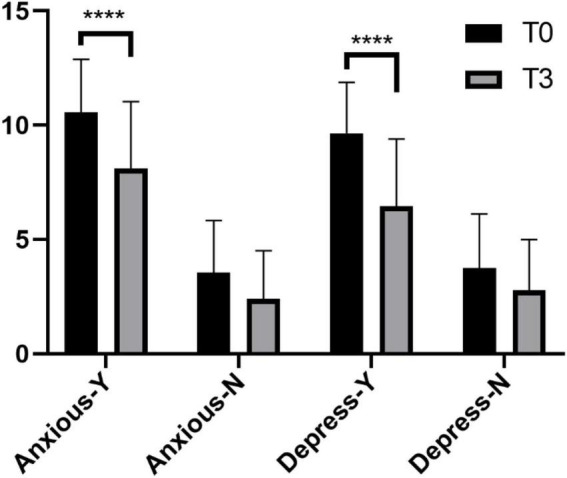
Changes in HADS scores before and six months after surgery. ****p < 0.001.

The number of patients with anxiety and depression decreased after either THA or TKA. However, the reduction was not significantly different (*p >* 0.05, Table 2). We analyzed the HADS scores of these patients at T0 and T3 (Figure 2), and the results showed that in patients who were anxious or depressed, there was a significant difference (*p <* 0.001) in the reduction of the T3 score compared with the T0 score. In non-anxious/depressed patients, the T3 scores were also decreased compared with the T0 scores, but the difference was not statistically significant (*p >* 0.05). This proves that TJA can reduce the degree of anxiety and depression in patients and reduce the number of patients with anxiety and depression to some extent.

**TABLE 2:** The changes in the patients’ levels of anxiety or depression.

Characteristic		Anxious		*P*-value	Depressed		*P*-value
		Y	N		Y	N	
Hip	T0	28	27	0.124	16	39	0.111
	T3	20	35		8	47	
Knee	T0	8	17	0.529	6	19	0.747
	T3	6	19		7	18	

*N: asymptomatic patients; Y: symptomatic patients.*

The results of this analytical separation between the number of anxious/depressed patients and their HADS scores may be because we only included 80 patients in this study, and the small sample size would have affected the statistics.

#### Clinical Outcomes

**FIGURE 3:**
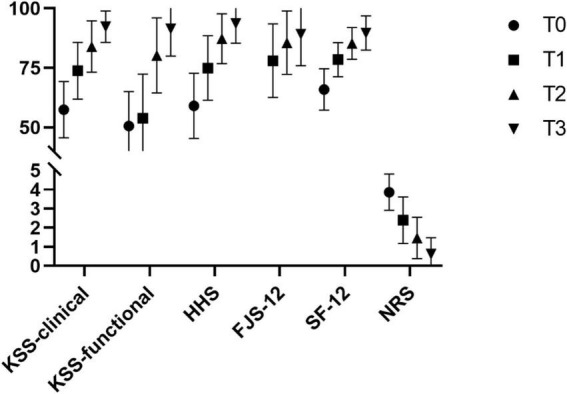
Evaluation index results before and after surgery.

Compared with the preoperative baseline scores, the HHS, KSS-C, KSS-F, SF-12, NRS, and FJS-12 scores at T3 increased significantly (*p <* 0.001), which suggests that arthroplasty surgery can significantly decrease pain and improve patients’ clinical outcomes and quality of life (Figure 3). After six months of follow-up, the satisfaction rate with joint arthroplasty, which was the primary outcome, was 77.5% (62/80).

### Psychological Health Effect

Using the preoperative HADS, we divided patients into the symptomatic or asymptomatic groups according to their anxiety and depression scores and evaluated rehabilitation (KSS, HHS, FJS-12, SF-12, and NRS scores) between the two groups at T0, T1, T2, and T3. The scores of the rehabilitation evaluation indicators in the different groups at each time point are shown in Table 3.

**TABLE 3:** The evaluation index results before and after surgery had categorized by the preoperative HADS score.

Characteristic	Anxious	T0	T1	T2	T3	Depressed	T0	T1	T2	T3
KSS-C	N	58.06 ± 10.37	79.18 ± 8.86	87.41 ± 9.20	95.18 ± 3.68	N	58.16 ± 9.78	78.11 ± 9.17	86.63 ± 8.98	94.79 ± 4.25
	Y	56.13 ± 15.20	62.38 ± 9.56	76.63 ± 86.25	86.25 ± 7.42	Y	55.17 ± 17.85	60.17 ± 9.50	75.50 ± 12.31	84.50 ± 6.75
KSS-F	N	55.59 ± 9.82	59.71 ± 17.27	86.18 ± 13.17	96.18 ± 5.46	N	54.47 ± 10.39	58.95 ± 16.46	86.05 ± 12.00	96.05 ± 54.00
	Y	40.00 ± 17.32	41.25 ± 15.53	67.50 ± 13.63	81.25 ± 14.33	Y	38.33 ± 37.50	37.50 ± 16.36	61.67 ± 9.31	76.67 ± 13.29
HHS	N	62.46 ± 10.52	80.72 ± 10.78	91.57 ± 6.18	96.16 ± 4.38	N	60.55 ± 13.59	75.71 ± 12.46	87.98 ± 7.83	94.61 ± 7.17
	Y	55.71 ± 15.68	69.39 ± 13.83	83.08 ± 11.98	91.30 ± 10.46	Y	55.29 ± 13.68	73.10 ± 16.27	85.47 ± 15.18	91.46 ± 10.69
FJS-12	N	–	85.64 ± 8.48	91.88 ± 7.28	94.93 ± 6.49	N	–	81.14 ± 12.81	88.46 ± 10.16	92.09 ± 9.84
	Y	–	68.66 ± 16.91	77.82 ± 14.88	82.05 ± 15.83	Y	–	69.71 ± 18.75	77.88 ± 17.28	81.35 ± 17.60
SF-12	N	70.07 ± 6.90	81.39 ± 3.27	87.64 ± 3.80	91.89 ± 4.71	N	68.05 ± 8.38	80.17 ± 4.20	86.50 ± 4.31	90.98 ± 5.10
	Y	60.89 ± 8.06	74.94 ± 8.94	82.39 ± 8.13	86.86 ± 8.55	Y	60.36 ± 7.03	74.05 ± 10.85	82.05 ± 10.01	86.05 ± 10.09
NRS	N	3.66 ± 0.94	1.84 ± 0.96	1.05 ± 0.91	0.30 ± 0.55	N	3.79 ± 0.97	2.28 ± 1.07	1.38 ± 1.02	0.43 ± 0.68
	Y	4.11 ± 0.92	3.06 ± 1.17	1.97 ± 1.06	1.00 ± 1.01	Y	4.05 ± 0.90	2.68 ± 1.52	1.68 ± 1.21	1.09 ± 1.11

*N: asymptomatic patients; Y: symptomatic patients.*

The results of the repeated measures multivariate analysis of variance were as follows (Table 4): (1) With the increase in time, the patients’ recovery improved, which was reflected by the scores of the KSS, HHS, FJS-12, SF-12, and NRS (all *p <* 0.001). (2) In the evaluation of the groups, the non-anxiety/depression group’s scores for the rehabilitation evaluation indicators were better than those of the anxiety/depression group at each time point (all *p <* 0.05), except for the HHS score. There was no difference between the non-depression group and the depression group (*p >* 0.05). (3) In the crossover analysis of group and time, the P-value of the KSS-C was less than 0.05, which suggested that patients without anxiety/depression had more significant and faster recovery than patients with anxiety/depression in the same time period. The SF-12 and NRS scores were also significant in anxious and non-anxious patients (*p <* 0.05), suggesting that non-anxious patients had faster and more effective recovery of life ability and pain relief than anxious patients at the same recovery time.

**TABLE 4:** Repeated measures multivariate analysis of variance results.

Characteristic		*P*-value					
		KSS-C	KSS-F	HHS	FJS-12	SF-12	NRS
Anxious	Group	0.003	<0.001	0.002	<0.001	<0.001	<0.001
	Time	<0.001	<0.001	<0.001	<0.001	<0.001	<0.001
	Group and Time	0.024	0.834	0.197	0.106	0.039	0.021
Depressed	Group	0.003	<0.001	0.235	0.001	<0.001	0.047
	Time	<0.001	<0.001	<0.001	<0.001	<0.001	<0.001
	Group and Time	0.048	0.618	0.702	0.879	0.220	0.374

When analyzing the satisfaction of patients six months after the operation, we divided the patients into four types: normal patients: A(–)&D(–); patients with anxiety only: A(+)&D(–); patients with depression only: A(–)&D(+); and patients with depression and anxiety: A(+) & D(+).

**FIGURE 4:**
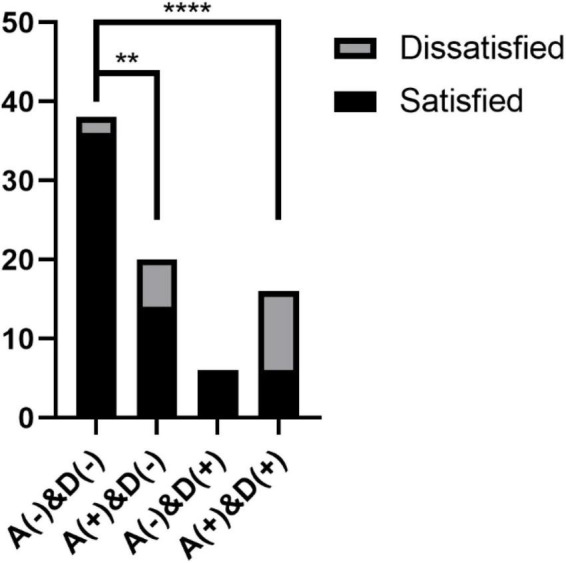
Postoperative satisfaction of patients: A(+): patients with anxiety; D(+): patients with depression; A(–): patients without anxiety; D(–): patients without depression. **p < 0.01, ****p < 0.001.

At T3, the patient satisfaction rate was 94.7% (36/38) for A(–)&D(–) patients, 70% (14/20) for A(+)&D(–) patients, 100% (6/6) for A(–)&D(+) patients, and 37.5% (6/16) for A(+)&D(+) patients (Table 5). The satisfaction rate of A(+)&D(–) patients and A(+)&D(+) patients was lower than the total satisfaction rate of 77.5% (62/80) and lower than that of A(–)&D(–) patients (*p <* 0.01 and *p <* 0.001, Figure 4).

**TABLE 5:** The number of patients who were satisfied or dissatisfied with the surgical results after 6 months.

Characteristic	A(−)&D(−)	A(+)&D(−)	A(−)&D(+)	A(+)&D(+)
Satisfied	36	14	6	6
Dissatisfied	2	6	0	10

*A(−): patients without anxiety; D(−): patients without depression.*

*A(+): patients with anxiety; D(+): patients with depression.*

These results suggest that the psychological state of patients has a significant impact on postoperative satisfaction and that the postoperative satisfaction of patients with anxiety and depression is significantly lower than that of normal patients. The satisfaction rate of patients with depression only seemed to be higher than that of normal patients, mainly considering the error caused by the small sample size.

### Factors Influencing Preoperative Psychological State

The effects of the independent variables (sex, BMI, age, course of the disease, SF-12 score at T0, NRS score at T0, HHS score at T0, and KSS score at T0) on the dependent variables (HADS score at T0 and SOC-13 score) were detected by multivariate linear regression (stepwise method).

In patients with hip diseases, the analysis indicated that the HADS-A score at T0 was correlated with the SF-12 T0 score and age (*R*^2^ = 0.472, *p <* 0.001, Supplementary Figure 1A), and the HADS-D score at T0 was correlated with the SF-12 score at T0 (*R*^2^ = 0.135, *p* = 0.006, Supplementary Figure 1B). In patients with knee diseases, the analysis showed that the HADS-A score at T0 and the HADS-D score at T0 were all correlated with the SF-12 score at T0 (*R*^2^ = 0.276, *p* = 0.007 and *R*^2^ = 0.195, *p* = 0.027, respectively; Supplementary Figures 2A,B).

**FIGURE 5:**
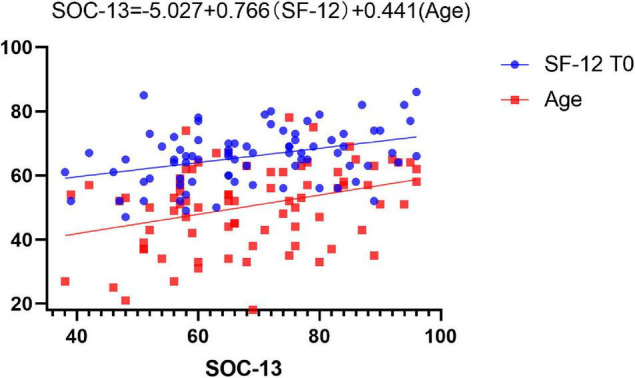
Influencing factors of the SOC-13 score.

The SOC-13 score was correlated with the SF-12 score at T0 and age (*R*^2^ = 0.308, *p <* 0.001, Figure 5). It shows that the SOC-13 score was affected by the SF-12 score and age, and it was positively correlated, indicating that a better quality of life and advancing age will increase patients’ confidence and ability to have a meaningful and manageable understanding of their lives.

### Factors Influencing Postoperative Psychological State

The effects of the independent variables (sex, BMI, age, course of disease, hospitalization time, intraoperative bleeding, length of surgery, SF-12 score at T3, NRS score at T3, FJS-12 score, HHS score at T3, and KSS score at T3) on the dependent variable (HADS score at T3) were detected by multivariate linear regression (stepwise method).

In patients undergoing THA, the analysis showed that the HADS-A score at T3 was correlated with the FJS-12 score at T3 and BMI (*R*^2^ = 0.603, *p <* 0.001, Supplementary Figure 3A), and the HADS-D score at T3 was correlated with BMI and the FJS-12 score at T3 (*R*^2^ = 0.599, *p <* 0.001, Supplementary Figure 3B). This finding indicated that the lighter the patient’s weight was, the higher the degree of joint amnesia and the better the postoperative psychological state.

In patients with TKA, the analysis suggested that the HADS-A score at T3 was correlated with the NRS score at T3 (*R*^2^ = 0.622, *p <* 0.001, Supplementary Figure 4A), and the HADS-D score at T3 was correlated with the KSS-F score at T3 (*R*^2^ = 0.609, *p <* 0.001, Supplementary Figure 4B). This finding indicated that the less pain the patient had, the higher their joint function, and the better their postoperative psychological status.

The above results suggest that the weight management and joint amnesia of patients after THA will have a significant impact on their psychological state after surgery. In patients after TKA, the factors that affect the postoperative psychological state are joint pain and joint function.

## Discussion

Our study aimed to investigate the influence of the psychological state on early-term clinical outcomes, patient satisfaction after TJA and the impact of postoperative recovery on psychological state. Our findings showed that the preoperative psychological state is not related to the severity of the disease, but the psychological state can affect postoperative recovery after TJA, and good functional rehabilitation after TJA can also effectively improve the patients’ psychological health.

Our study shows that the scores of the postoperative evaluation indices were influenced by the patients’ psychological health states (Table 4). From our analysis, we found that a poor psychological state was negatively related to early postoperative recovery, which is in line with published studies (8, 9, 12, 43). Anxiety and depression symptoms led to lower satisfaction rates of patients at 6 months postoperatively (Table 5).

Our study analyzed the relationship between preoperative psychological status and preoperative measures and showed that only the SF-12 score and age could affect the HADS and SCO-13 scores (Figure 5 and Supplementary Figures 1, 2).

The analysis between postoperative psychological status and the postoperative measures showed that in patients undergoing THA, their FJS-12 score and BMI were negatively correlated with their postoperative psychological state; that is, the lighter the patient’s weight, the higher the degree of joint amnesia, and the better the postoperative psychological state (Supplementary Figure 3). In TKA patients, the patients; NRS and KSS-F scores were correlated with postoperative psychological status; that is, the less pain the patient had, the higher the joint function, and the better the postoperative psychological status (Supplementary Figure 4).

In contrast with patients undergoing THA, the postoperative recovery of patients undergoing TKA was more affected by psychological factors, such as in the evaluation of the group. The KSS score of the non-anxiety and depression groups at each time point was better than that of the anxiety and depression groups (*p <* 0.05), but there was no significant difference in the HHS (*p >* 0.05, Table 4). In the crossover analysis of group and time, the *p*-value of the KSS-C score was less than 0.05, which suggested that patients without anxiety/depression had more significant and faster recovery than patients with anxiety/depression in the same time period, but there was no significant difference in the HHS (*p >* 0.05, Table 4).

In summary, our study found that the preoperative psychological state influenced postoperative recovery, and better postoperative recovery (FJS-12 score in THA patients, NRS and KSS-F scores in TKA patients) was positively correlated with improvements in anxiety and depression. These findings provide a strategy for us to effectively improve the postoperative satisfaction and postoperative recovery of patients. First, before TJA (especially TKA), we identified patients who were in a poor psychological state. Then, drugs or psychological counseling were used to improve the psychological state of these patients so that they had enough confidence to perform rehabilitation exercises after the operation. Sufficient rehabilitation exercises enable patients to achieve good functional recovery of the affected limbs, which is conducive to the improvement of the patients’ anxiety and depression and increases the patients’ satisfaction. After this, a closed loop of positive feedback is formed.

This study has several limitations. First, the sample size was small, and it only reflected one institutional database. Second, this study only looked at short-term outcomes; medium- and long-term outcomes should be studied further. Third, other variables, such as disease severity or comorbidities, may affect postoperative recovery, but due to insufficient information, we were unable to analyze these factors. Therefore, we only analyzed patients with successful TJA without complications. In the future, we will conduct a study on psychological interventions in patients with anxiety and depression who are undergoing TJA.

## Conclusion

Finally, the psychological state can affect recovery after TJA, and successful TJA can help improve patients’ psychological states, especially after TKA.

## Data Availability Statement

The raw data supporting the conclusions of this article will be made available by the authors, without undue reservation.

## Ethics Statement

The studies involving human participants were reviewed and approved by the Ethics Committee of Central South University Xiangya Hospital. The patients/participants provided their written informed consent to participate in this study.

## Author Contributions

ZZ, QX, and LW conceptualized the study. ZZ and QX analyzed and interpreted the data. ZZ was drafted the manuscript. LW and YH revised the manuscript. All authors contributed to the data collection and organization, read and approved the final manuscript.

## Conflict of Interest

The authors declare that the research was conducted in the absence of any commercial or financial relationships that could be construed as a potential conflict of interest.

## Publisher’s Note

All claims expressed in this article are solely those of the authors and do not necessarily represent those of their affiliated organizations, or those of the publisher, the editors and the reviewers. Any product that may be evaluated in this article, or claim that may be made by its manufacturer, is not guaranteed or endorsed by the publisher.
