# Supplementary material for: The Impact of Psychological Health on Patient Recovery After Arthroplasty
Source: Front Psychiatry. 2022 Jun 30;13:817716. doi: 10.3389/fpsyt.2022.817716 (PMC9279863; doi:10.3389/fpsyt.2022.817716)

Supplementary figure 1. Factors influencing the preoperative psychological state in patients with hip diseases. (a: HADS-A score at T0; b: HADS-D score at T0.)

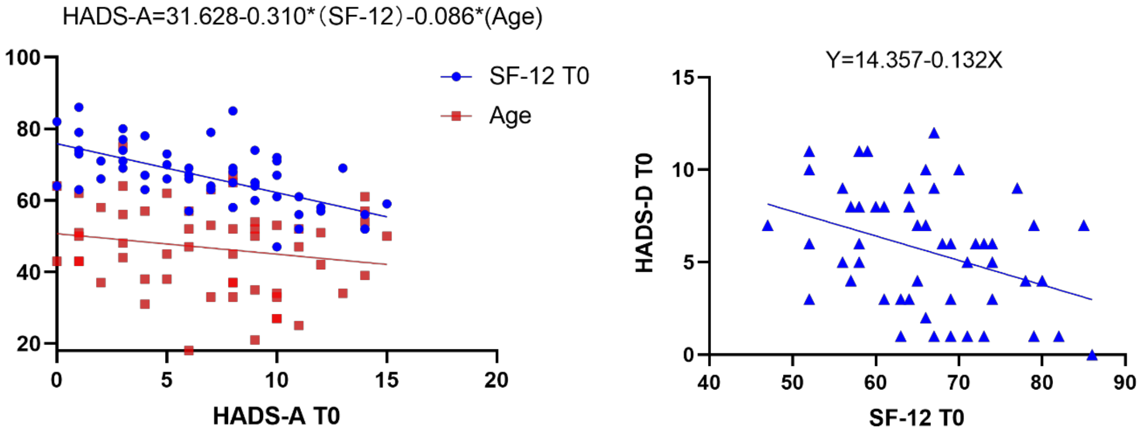

Supplementary figure 2. Factors influencing the preoperative psychological state in patients with knee diseases. (a: HADS-A score at T0; b: HADS-D score at T0.)

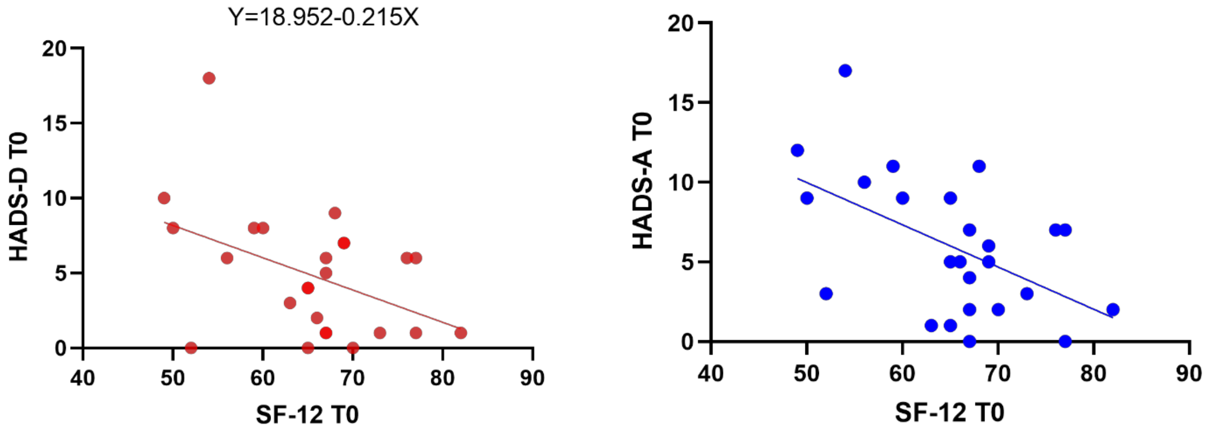

Supplementary figure 3. Factors influencing postoperative psychological state in patients with hip diseases. (a: HADS-A score at T3; b: HADS-D score at T3.)

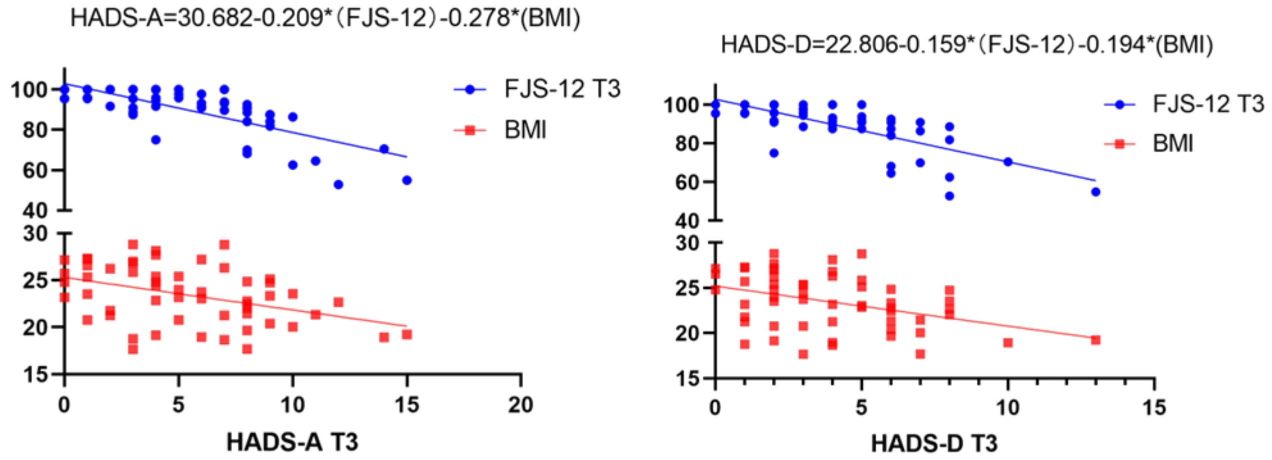

Supplementary figure 4. Factors influencing postoperative psychological state in patients with knee diseases. (a: HADS-A score at T3; b: HADS-D score at T3.)

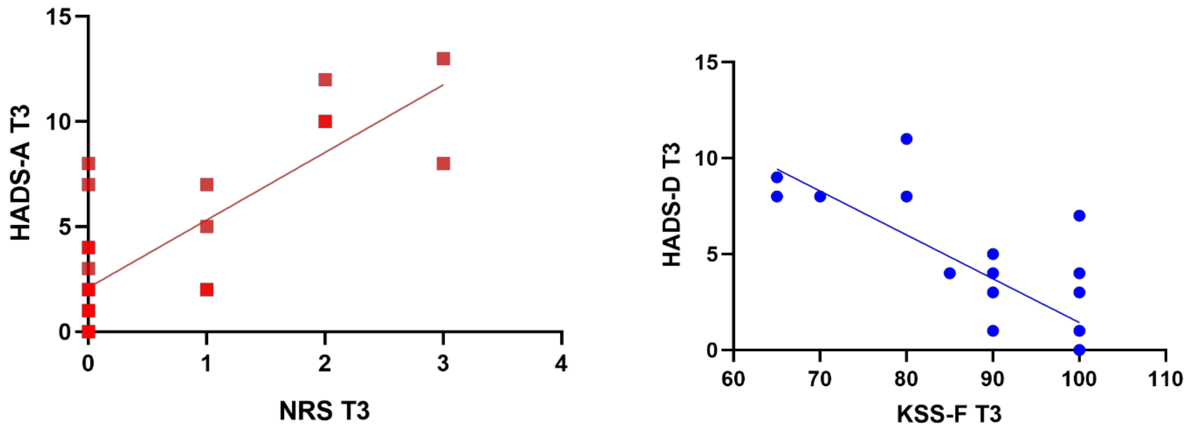

Supplement: Supplementary file 1 [file Data_Sheet_1.pdf]
